# Cloning, Exogenous Expression and Function Analysis of Interferon–γ from *Gadus macrocephalus*

**DOI:** 10.3390/v14102304

**Published:** 2022-10-20

**Authors:** Jielan Jiang, Jie Gu, Aijun Zhan, Mingguang Mao, Yumeng Liu, Haishan Wang, Yunxiang Mao

**Affiliations:** 1 Yazhou Bay Innovation Institute, Hainan Tropical Ocean University, Sanya 572022, China; 2 Key Laboratory of Utilization and Conservation for Tropical Marine Bioresources, Ministry of Education, Hainan Tropical Ocean University, Sanya 572022, China; 3 College of Fisheries and Life Science, Dalian Ocean University, Dalian 116023, China; 4 Shenzhen Academy of Inspection and Quarantine, Shenzhen 518010, China

**Keywords:** interferon γ, prokaryotic expression, yeast expression, *Gadus macrocephalus*

## Abstract

Interferon γ (IFN–γ) is now considered to be one of the key molecules in the regulation of innate and adaptive immunity. The function of IFN–γ is best described in humans, but less of IFN–γ in fish species has been described at protein level. In the present study, IFN–γ from *Gadus macrocephalus* (GmIFN–γ) has been examined in terms of bioinformatics, prokaryotic expression, yeast expression, antiviral activity and immune regulatory function. The cDNA of GmIFN–γ contains an open reading frame of 570 nucleotides, coding 189 amino acids. The mature protein contains a nuclear localization signal motif and an obvious IFN–γ signature sequence at the C-terminal. GmIFN–γ is very similar to that of Atlantic cod, with homology up to 89.89%, but less than 32% to other species. GmIFN–γ can be detected in the gills, spleen, intestine, brain and kidney. Interestingly, during early development, a strong signal of GmIFN–γ was not detected until 40 days post hatching. Prokaryotic expression plasmid pET–32a–GmIFN–γ was constructed, and the expression products in BL21 were confirmed by Mass Spectrometry. Meanwhile, the plasmid pGAPZA–GmIFN–γ with Myc tag was constructed and transmitted into *Pichia pastoris* yeast GS115, and the products were tested using Western blot. The purified GmIFN–γ from either BL21 or yeast has a strong antivirus (Spring viremia of carp virus) effect. The vector of *pcDNA3.1*–GmIFN–γ was expressed in EPC cell lines; high transcript levels of MHC class I chain-related protein A (MICA) gene were detected; and the exogenous *Gm*IFN–γ protein could also induce MICA expression, indicating that GmIFN–γ could stimulate immune response. The yeast GS115 with *Gm*IFN–γ protein, which is an inclusion body, was given to zebrafish orally, and the transcript of zebrafish *IFN–γ* was upregulated significantly; however, genes of the interferon type–I signal pathway were not well stimulated.

## 1. Introduction

Interferon γ (IFN–γ) is the only member of type II cytokines in mammals and plays a significant role in cell defense against intracellular pathogens. Unlike humans with only one IFN–γ, some fish species have a broader type II interferon family, consisting of two members, IFN–γ and fish-specific IFN–γ related (IFN–γrel) proteins. Comparative studies of duplicated copies of IFN–γ were performed for Atlantic salmon (*Salmo salar*) [1], common carp (*Cyprinus carpio*) [2] and rainbow trout (*Oncorhynchus mykiss*) [3]. However, only a few of the IFN–γ genes in fish have been described at protein level. The Fugu IFN–γ protein (UniProtKB—Q708J2) is expressed as a 189 amino-acid-long preprotein with a signal peptide 22 amino acids long and one potential glycosylation site, similar to rainbow trout (188 aa, AT rich) [4].

The cellular response in fish activated by IFN–γ is composed of a tetrameric complex formed by IFN–γR1, which is mainly responsible for binding ligands, and IFN–γR2, which is mainly responsible for transmitting cellular signals [5]. IFN–γR1 and IFN–γR2 recruited Jak1 and Jak2, respectively, to the cytoplasmic domain, and when activated by IFN–γ homodimers, they subsequently caused signaling and phosphorylation of signal transducers and activators of transcription 1 (Stat1). Phosphorylated Stat1 homodimers migrate to the nucleus and bind to gamma IFN activation sites (GAS) in the promoter region of IFN–γ effector genes and initiate the expression of effector genes [6].

*Gadus macrocephalus*, as an important fishery species in the North Pacific, is now under consideration as an aquaculture species in China. However, diseases inhibit artificial breeding, especially the nervous necrosis virus (NNV), belonging to the BFNNV genotype. We try to explore some antivirus proteins or immune factors, including the interleukins, INF–α and IFN–γ, and then apply them in cod larvae via an oral route. In this study, we aimed to clone IFN–γ (GmIFN–γ) from *G. macrocephalus;* to construct kinds of expression vectors to get GmIFN–γ protein; and to examine its antivirus and immunoregulatory activities.

## 2. Materials and Methods

### 2.1. Fish, Cell Culture, Virus and Reagents

Several adult *G. macrocephalus* were captured from the Yellow Sea near Lvshun, Dalian City. Tissues of the gill, brain, muscle, kidney and spleen were sampled for RNA extraction. Five whole larvae at 5, 10, 20, 25, 30 and 45 days post hatching (dph) were sampled, respectively. Epithelioma papulosum cyprinid (EPC) cells were provided by Prof. Qiya Zhang from the Chinese Academy of Sciences and cultured at 25 °C in medium 199 with 10% fetal bovine serum (FBS). Spring viremia of carp virus (SVCV) was offered by Shenzhen Customs, and it can infect the EPC cell line. All animal protocols required for this research were approved by the Animal Care and the Biosafety Committee at Yazhou Bay Innovation Institute, Hainan Tropical Ocean University.

### 2.2. Molecular Cloning and Sequence Analysis of GmIFN–γ

Total RNA of the different organs was extracted using Trizol (Sangon Biotech, Shanghai, China). First, 1 µg of RNA was used to obtain cDNA using the Superscript Preamplification System (TaKaRa). Primers (*IFN–γ*f1 and *IFN–γ*r1) for GmIFN–γ cloning were designed based on the previous transcriptome sequences [7] (Table 1). The open reading frame (ORF) of GmIFN–γ was obtained using PCR. The purified PCR products were then subcloned into vector pMD–18T and sequenced by TSINGKE Biological Technology (Guangzhou, China). The nucleotide sequence was compared to the GenBank/EMBL databases using BLAST from the National Center for Biotechnology Information (NCBI) (http://www.ncbi.nlm.nih.gov/blast). Multiple sequence alignments were performed with DNAMAN V6 (Lynnon). Molecular weight was calculated using Expasy ProtParam (https://web.expasy.org/protparam). The signal peptide was analyzed using the SignalP–5.0 Server (http://www.cbs.dtu.dk/services/SignalP/). The functional domains were predicted by gene structure analysis using the software IMGT (http://www.imgt.org). The N–glycosylation sites were analyzed with the NetNGlyc 1.0 server (http://www.cbs.dtu.dk/services/NetNGlyc/). A 3D model of *Gm*IFN–γ was predicted using SWISS–MODEL (http://swissmodel.expasy.org); images were edited using Raswin (Windows Version 2.7.5.2).

### 2.3. Expression Pattern of GmIFN–γ in G. macrocephalus

Specific primers (*IFN–γ*f2 and *IFN–γ*r2) of GmIFN–γ were designed for qPCR, and primers (*ActinF1* and *ActinR1*) of *β*–actin as housekeeping gene were designed according to our previous study [8]. Triplet repeats were set for each qPCR test. The transcript levels of target genes were calculated relative to *β–actin* using the 2^−ΔΔCT^ method.

### 2.4. Preparation of Recombinant Plasmid

Specific primers with restriction enzyme site were designed for plasmid construction (Table 1). The recombinant plasmid vectors *pET32a–*GmIFN–γ, *pGAPZA–*GmIFN–γ and *pcDNA3.1–*GmIFN–γ have been prepared by subcloning, according to the previous work [9]. The products were sequenced by TSINGKE Biological Technology (Guangzhou, China). *pET32a–*GmIFN–γ was used for prokaryotic expression, and *pGAPZA–*GmIFN–γ was used for yeast expression. *pcDNA3.1–*GmIFN–γ was transmitted into EPC cell for function analysis.

### 2.5. Prokaryotic Expression and Purification

pET32a–GmIFN–γ was transmitted into *E. coli* BL21(DE3), which was cultured in LB medium with 1 mM isopropyl β–D–thiogalactoside (IPTG) at 37 °C. Expression of the pET32a–GmIFN–γ was induced using 1.0 mM IPTG at 37 °C. After centrifugation at 12,000× *g* rpm at 4 °C, bacterial suspension was collected at 1, 2, 3, 4, 5 and 6 h, respectively, for further detection by SDS–polyacrylamide gel electrophoresis (SDS–PAGE). The soluble histidine (His)-tagged fusion *Gm*IFN–γ was further purified using Ni–NTA affinity column (Sangon, Shanghai), according to the manufacturer’s instructions. The purified protein was sent to Sangon Biotech and verified by Mass Spectrometry (MS).

### 2.6. Yeast Expression and Isolation of GmIFN–γ

The plasmid pGAPZA–GmIFN–γ was linearized with BspH1 restriction enzyme. When necessary, the plasmid was concentrated and purified using the isopropanol method [10]. The linearized pGAPZA–GmIFN–γ was transformed into *Pichia pastoris* yeast GS115, following the instructions of the Quick & Easy Yeast Transformation Mix kit (TaKaRa). Yeast recombinants were screened using different concentrations of Bleomycin (Thermo Fisher) YPD (Yeast Peptone Dextrose) plates to select the positive yeast strains with high copy of GmIFN–γ gene. The positive strain was cultured in fresh YPD medium for continued cultivation. Yeasts were sampled and centrifuged at 24, 72 and 96 h, respectively, and the protein was extracted using the One Step Yeast Active Protein Extraction kit (Sangon), according to the manufacturer’s instructions. Western blot was performed to identify the isolated protein. The detection antibodies used in the experiment were Anti-Myc tag mouse monoclonal antibody (Order NO. D191042, Sangon Biotech) and HRP-conjugated Goat Anti-Mouse IgG (Order NO. D110087, Sangon Biotech).

### 2.7. Antivirus Effects of GmIFN–γ

GmIFN–γ from *E. coli* or yeast were evaluated for their antiviral function. In the experiment, the GmIFN–γ protein was diluted to 100 μg/mL, and 100 μL was added to EPC cell lines, which were cultured to approximately 100% confluency. After that, 100 μL of SVCV (titer 2.73 × 10^−7^ PFU/mL) was inoculated onto the cells. The negative group was not treated with protein or inoculated with the virus, while the positive group was not treated with protein but was inoculated with SVCV. Each group was performed in triplicate. MEM (3% methylcellulose; 10% FBS; 2% Pen–Strep (GIBCO 600-5140)) was added to each well of the 24-well plate in a constant temperature incubator at 25 °C for 4–5 d, and the formation of plaques after staining with crystal violet was observed [11].

### 2.8. Immunoregulation of GmIFN–γ in the EPC Cell Line

GmIFN–γ from *E. coli* or *P. pichia* was diluted to 100μg/mL with PBS, then added into EPC cell line for 24 h. pcDNA3.1–GmIFN–γ was transmitted into EPC cells using FuGENE 6 Transfection Reagent (Promega) and Opti-MEM (GIBCO), according to the manufacturer’s instructions, and cells were sampled after 24 h. Each group was performed in triplicate. RNAs were extracted from each group, and cDNAs were synthesized as PCR templates. Primers (MicaF and MicaR) based on the MHC class I chain-related protein A (MICA) gene (GenBank No. XM_042729996) were used to valuate cell response to GmIFN–γ. Carp *β–actin* (ActinF2 and AcinR2) was the housekeeping gene. The qPCR procedure was performed according to Section 2.3.

### 2.9. Zebrafish Immune Response to GmIFN–γ by Oral Route

The zebrafish (aged 2–4 weeks) were randomly divided into two groups, with 25 tails in each group, and were kept in a 57 × 39 × 31 cm water tank for 5 d. The control group was set with 1 g *P. pichia* GS115 feeding per day, and the experimental group was fed with 1 g of *P. pichia* GS115, which expressed GmIFN–γ as inner cellular protein. The experiment lasted for 5 d, and three zebrafish were randomly sampled every 24 h from each group. The viscera were sampled for gene detection. Genes from interferon signal pathway including *PKR*, *Mx*, *IRF1*, *IFN–γR1*, *IFN–γ1* and *IRF7* were tested using relative qPCR (primers were shown in Table 1), and *β–actin* (ActinF3 and AcinR3) was used as the housekeeping gene.

## 3. Results

### 3.1. Bioinformatic Analysis of GmIFN–γ

**Figure 1:**
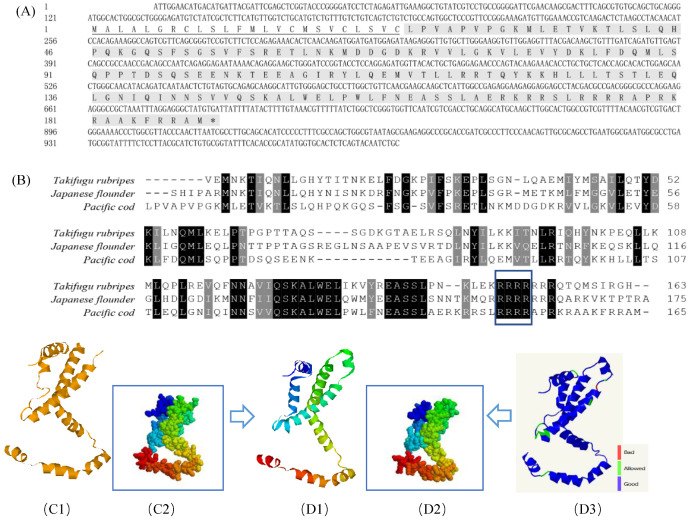
Bioinformatic analysis of GmIFN–γ gene. (**A**) cDNA and the deduced amino acid of GmIFN–γ. Signal peptide was underlined. (**B**) Mature IFN–γ peptides among *Takifugu rubripes*, Japanese flounder and *G. macrocephalus* were aligned. Identical or similar amino acids are shaded (black: present in all species; dark gray: present in 80% of the species). The nuclear localization signal and the putative IFN–γ signature sequence are boxed in green. (**C1**–**D3**): 3D models of IFN–γ compared between Japanese flounder and *G. macrocephalus*. (**C1**,**C2**): a 3D model of Japanese flounder displayed in Ribbons and Spacefill style, separately. PDB code is c6f1eA. (**D1**,**D2**): a 3D model of GmIFN–γ was constructed based on the template c6f1eA. Both were displayed in Ribbons and Spacefill style, separately. (**D3**) Secondary structure assignment was carried using PROCHECK. Good quality is indicated in blue, while the bad quality is indicated in red.

The nucleotide sequences of GmIFN–γ have been deposited to the GenBank with accession numbers of OK485132. The ORF of GmIFN–γ encoded a 165 amino acid (aa) mature peptide and a predicted 24-aa signal peptide sequence (Figure 1A). The mature protein contains a nuclear localization signal (NLS) motif and an obvious IFN–γ signature sequence at the C-terminal (Figure 1B). A 3D model of the mature GmIFN–γ was constructed based on the template of the Japanese flounder IFN–γ (Figure 1C,D).

**Figure 2:**
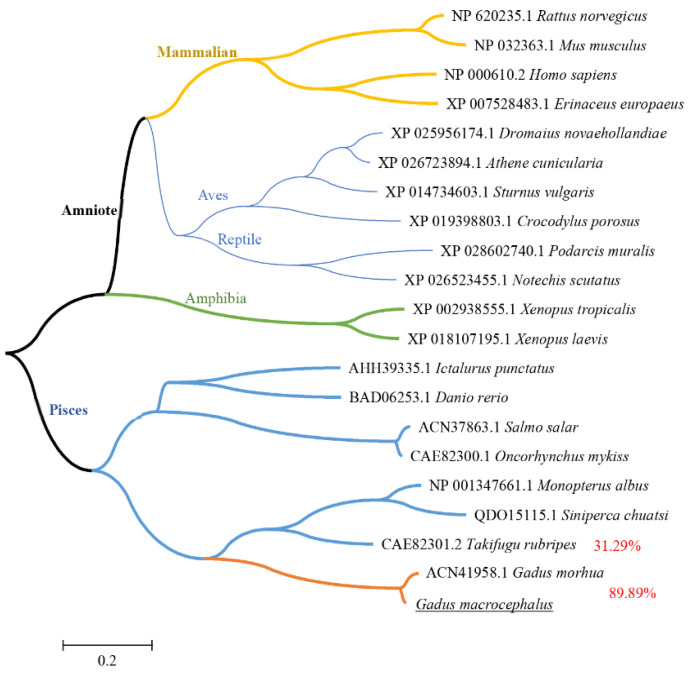
Phylogenetic tree of vertebrate IFN–γ peptides. The trees were computed using Neighbor-Joining method by MEGA version 5.0. Different levels of evolution were highlighted in various color.

Sequences of *IFN–γ* from cod and other species were gathered for multiple alignment by the ClustalW program. Phylogenetic trees were shown in Figure 2. GmIFN–γ is very similar to that of Atlantic cod, with homology up to 89.89%, but less than 32% to other species, indicating that GmIFN–γ may have unique features.

### 3.2. Expression Profiles of GmIFN–γ

**Figure 3:**
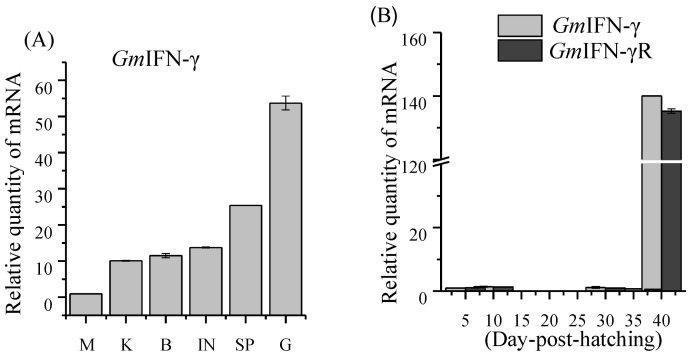
Expression profiles of GmIFN–γ. (**A**) Expression levels of GmIFN–γ in adult Pacific cod muscle (M), kidney (K), brain (B), intestine (I), spleen (SP) and gill (G). (**B**) Expression pattern of GmIFN–γ and its receptor in cod larvae during the early developmental stages.

As shown in Figure 3A, GmIFN–γ can be detected in all tissues and mainly expressed in the immune related tissues, such as the gills and intestine. Interestingly, GmIFN–γ was at a relatively low level before 35dpf but increased significantly after 40 dpf (Figure 3B). The expression pattern of GmIFN–γ receptor (GmIFN–γ*R*) was similar to that of GmIFN–γ. The result indicated that GmIFN–γ expression is related to the development time.

### 3.3. Exogenous Expression of GmIFN–γ

**Figure 4:**
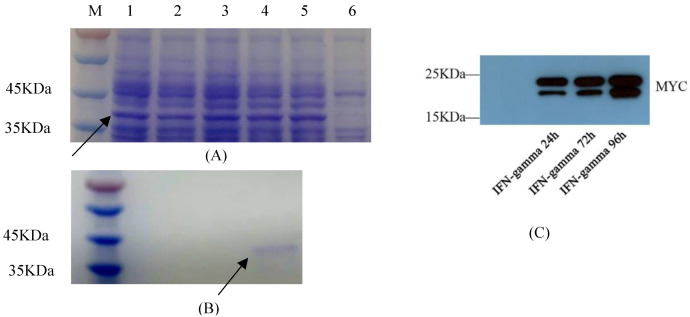
Exogenous expression of GmIFN–γ. (**A**) Optimization of prokaryotic expression conditions. Nos. 1–5 indicate that the protein was induced for 1–5 h. No. 6 was set as the control. Target protein was marked by arrow. (**B**) Purification of GmIFN–γ from *E. coli*. Target protein was marked by arrow. (**C**) GmIFN–γ expressed in yeast GS115 and detected using Western blot at 24, 72 and 96 h, respectively.

Prokaryotic expression vector pET-32a-GmIFN–γ was constructed with a 6 × His tag, and the expression conditions were optimized. The target protein was induced with 1 mM IPTG for 4 h at 37 °C to obtain the highest yield, in the form of inclusion bodies and monomers (Figure 4A). After purification, the protein concentration was 216 μg/mL. The products were confirmed by MS (Supplementary Table S1).

*P. pichia* GS115 was sampled at 24 h, 72 h and 96 h, and GmIFN–γ protein was detected using Western blot (Figure 4C). There are two bands in the figure. One is near 21 kD, and another is near 22 kDa (Figure 4C). It was considered that part of the target protein in the yeast was modified by N-glycosylation, leading to a larger molecular.

### 3.4. GmIFN–γ Inhibits SVCV Growth

**Figure 5:**
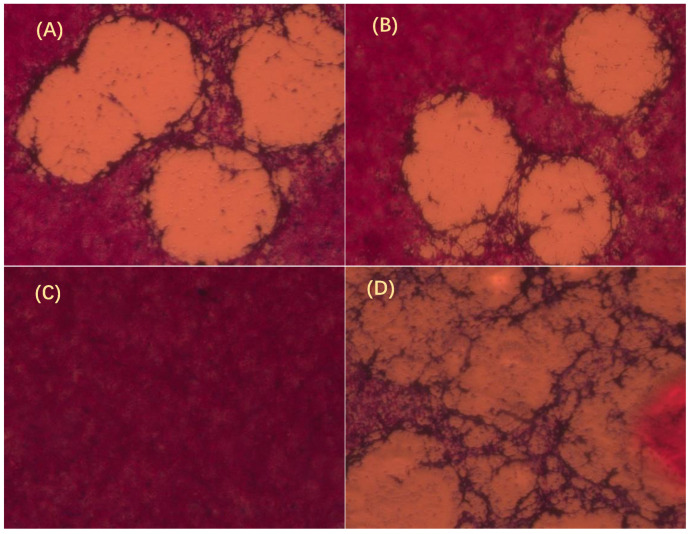
GmIFN–γ inhibits Spring viremia of carp virus (SVCV) growth observed by plaque assay. (**A**) GmIFN–γ from yeast expression. (**B**) GmIFN–γ from prokaryotic expression. (**C**) EPC was used as a negative control. (**D**) EPC was infected by SVCV as a positive control. Live cells were stained red with neutral red indicator. The concentrations of purified GmIFN–γ from yeast expression and prokaryotic expression were both 100 μg/mL.

SVCV was cultured in EPC cells, and GmIFN–γ protein was added into the cell line. Compared with the control (Figure 5C,D), growth of SVCV was inhibited significantly by GmIFN–γ, whether it was from yeast or *E. coli* (Figure 5A,B).

### 3.5. MICA Expression in EPC Induced by GmIFN–γ

**Figure 6:**
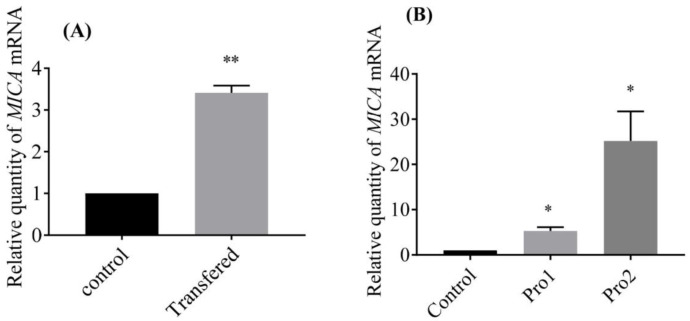
*MICA* was induced by GmIFN–γ. (**A**) pcDNA3.1-GmIFN–γ was transferred into EPC cells, and the endogenous GmIFN–γ induced the MICA expression; ** indicates *p* < 0.01. (**B**) EPC cells were incubated with 100 μg/mL GmIFN–γ for 24 h. Pro1 was from *E. coli* BL21, while Pro2 was from *P. pastoris* GS115; * indicates *p* < 0.05.

*MICA* of EPC cell was up-regulated significantly when pcDNA3.1-GmIFN–γ was transferred into EPC cell line for 24 h (Figure 6A). On the other hand, *MICA* was also upregulated when cells were treated with isolated GmIFN–γ protein. The level of *MICA* induced by yeast-expressed GmIFN–γ was significantly higher than that induced by *E. coli-*expressed GmIFN–γ (Figure 6B).

### 3.6. Response of Zebrafish Interferon System to Yeast Expressed GmIFN–γ

**Figure 7:**
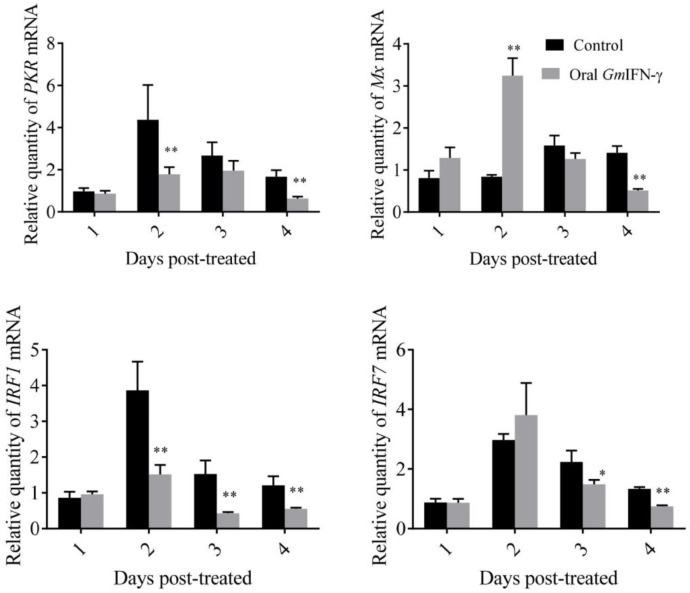

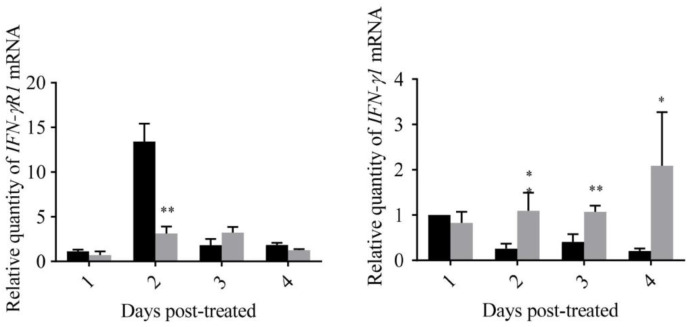
Expression levels of genes related to the IFN–I signal pathway of zebrafish fed with yeast containing GmIFN–γ. T tests were used for statistical analysis. * indicates significant differences (*p* < 0.05), while ** indicates strong significant differences (*p* < 0.01).

pGAPZA is a well-known intracellular expression vector, and the target protein is usually expressed as inclusion bodies in yeast. Zebrafish were fed with yeast containing GmIFN–γ for several days. The mRNA level of IFN-related genes was detected using qPCR. It was shown that *IRF1*, *PKR* and *IFN–γR1* were down-regulated sometimes, while the expression of *Mx* was up-regulated two days later. More interestingly, the endogenous *IFN–γ1* of zebrafish was significantly up-regulated in the experiment (Figure 7). It was suggested that GmIFN–γ could not stimulate IFN–I signal pathway effectively in zebrafish, but it could evoke zebrafish IFN–γ1 expression obviously.

## 4. Discussion

### 4.1. Evolution and Conservation of GmIFN–γ Protein

Phylogenetic tree revealed that GmIFN–γ is quite different from other fish species except *Gadus*. The evolution feather is very similar to its receptor GmIFN–γR (data were shown in Figure S1). However, the conserved structure indicates that GmIFN–γ may share the similar function to other fishes. According to the sequence alignment, C–terminal of the *IFN–γ* is more conserved than the N–terminal. It was reported that the absence of varying degrees of C–terminal NLS region will make IFN–γ inactive and unable to resist virus invasion, which was verified by research on the effects on A549 cells against encephalomyocarditis virus, by a variety of human IFN–γ fusion proteins [12]. The IFN–γ recombinant protein of rainbow trout lacking NLS could not activate macrophages and induce the expression of immune genes such as γIP10 and STAT1 signal transduction molecules [13]. Additionally, the structure of *IFN–γ* receptor needed to be taken into account, as it is the key structure used to receive the signal of INF–γ. Unfortunately, IFN–γ receptors are quite different among various species (data were shown in Figure S1), so we have to confirm the function of GmIFN–γ in various aspects.

### 4.2. Expression of GmIFN–γ In Vivo and In Vitro

In vivo, GmIFN–γ mainly expresses in gills, which are the first protective barrier of fish, indicating that GmIFN–γ plays roles in immune defense. Interestingly, the transcript level of GmIFN–γ was very low before 35 dpf, and it is suggested that GmIFN–γ depends on the development of immune organs. Our previous work has elucidated that recombination activation gene 1 (Rag1), as one of the key genes in adaptive immune system, generally expressing around 60 dpf in *G. macrocephalus* [7]. So this result indicates that GmIFN–γ, as an innate immune factor, will work before T or B cell maturing, and its expression will be independent of the adaptive immune system.

In vitro, we have constucted several expression vectors for further study. Even though exogenous GmIFN–γ has been obtained from *E. coli* or *P. pastoris,* we do not know how its structure is modified in the *E. coli* or *P. pastoris*; thus, we do not know whether the products work or not. We believe that the eukaryotic expression system will be more credible for protein function. In yeast expression system, two bands were detected in the products using WB. It was reported that *P. pastoris* has the capability of N–glycosylation [14,15]. Glycosylation of protein will result in two bands in WB [16]. Glycosylation is one of the most important modifications after protein translation, involving cellular immunity, protein translation regulation and protein degradation [17,18].

### 4.3. Function analysis of GmIFN–γ

It was reported that some antiviral effector genes, such as guanylate binding protein 1 (GBP1) gene, Mx gene and interferon stimulated gene 15 (ISG15), as well as genes related to the activation of NADPH oxidase, can be induced by IFN–γ [19,20]. In the present study, Mx gene can be up-regulated by oral GmIFN–γ. However, PKR, IFR1 and IRF7 were down-regulated. Interestingly, the transcript level of zebrafish IFN–γ was significantly up-regulated. Some studies have found that crucian carp IFN can induce the expression of its own genes through the Stat1 pathway, forming a positive feedback loop [21].

Different types of interferon induce cells to synthesize antiviral proteins at different times. It was found that a type I interferon usually produces antiviral protein after 18 h of treatment of cells, while type II interferon usually produces related proteins after 24 h of treatment [22]. Therefore, in this experiment, GmIFN–γ was co-cultured with cells for 24 h, and then the virus was introduced. In the study, recombinant GmIFN–γ has an anti-SVCV effect, offering us a potential antivirus peptide for further application in cod larvae.

MHC molecules play a central role in immune response and non-self-recognition [23]. Among them, fish MHC I molecules are generally distributed on the surface of all nucleated cells [23,24,25], and they mainly express endogenous pathways. Their main function is to bind to endogenous protein polypeptides, such as viral protein, and present them to CD8+ cells and activated cells. In the experiment, the expression level of *MICA* of EPC was tested to valuate the immunomodulatory effect of GmIFN–γ, and it was up-regulated significantly. The increased expression of *MICA* indicates that IFN–γ plays an important role in the process of antigen presentation [26].

We believe that food chain will be an excellent way to apply medicine in fish larvae or juveniles. To our knowledge, this is the first time that IFN–γ was passed from yeast to fish by oral route, and it works. There is no doubt that it is an innovative method of medicine application in fish larvae or juveniles, but more details need further study, depending on different target medicine, such as vaccine, antibacteria peptides and antivirus peptides.

## Figures and Tables

**Table 1:** Sequence of primers used in this experiment.

Name	5′-3′Primer Sequence	Purpose
*IFN–γ*f1 *IFN–γ*r1	GAAAGGCTGTATCGTCCTGCCGGGG TGAACCACCCGAGCCAGATAAAAAC	Cloning of *GmIFN–γ*
*IFN–γ*f2 *IFN–γ*r2	CAAGACTCTAAGCCTACAACA ACACCTTCCCAAGCACAA	*qPCR for GmIFN–γ*
ActinF1 ActinR1	CCAAAGCAACAGGGAGA GCAGTGGTGGTGAAGGAGTAG	Internal control gene of qPCR in *Gadus macrocephalus*
ActinF2 ActinR2	GGCACTGCTGCTTCCTC ACCGCAAGACTCCATACCC	Internal control gene of qPCR in EPC cell
*pF1* *pR1*	CGGAATTCCTGCCAGTGGCTCCCGTT CCGCTCGAGCATAGCCCTCCTAAATTT	Plasmid construction of *pET32a–GmIFN–γ*
pF2 pR2	CGGAATTCATGCTGCCAGTGGCTCCCGTTC CCGCTCGAGGTCATAGCCCTCCTAAATTTAG	Plasmid construction of *pGAPZA*–*GmIFN–γ*
pF3 pR3	CCGCTCGAGATGCTGCCAGTGGCTCCCGTTC CGGAATTCGTCATAGCCCTCCTAAATTTAG	Plasmid construction of *pcDNA3.1*–*GmIFN–*γ
MicaF1 MicaR1	GAGATTCTGCCCAACGG CAGCCACATCTGAAACAAA	qPCR of *MICA* in EPC cell
ActinF3 ActinR3	CATGTTCGAGACCTT AGGCAGCTCATAGCT	Internal control gene of qPCR in *D. rerio*
PkrF PkrR	ACCAAACCCAGCAAAGG AGAAGTAGCGGACGATG	qPCR of *PKR* in *D. rerio*
MxF MxR	TGGCTGGAGCAGGTGTT AATGCTTCTGTGGTGGC	qPCR of *Mx* in *D. rerio*
Irf1F Irf1R	ATGCCCGTGTCCAGAAT CTGAGTCACTCCCTCCTTAT	qPCR of *IRF1* in *D. rerio*
IfnrF1 IfnrR1	GTCCAATCGCAATCCAC CCGTAAGCGTCTACAATA	qPCR of *IFN–γR1* in *D. rerio*
IrfF1 IrfR1	CAGAATGACAGCGTGGAT CTTTAGCCTGCCGTCTC	qPCR of *IFN–γ1* in *D. rerio*
Irf7F Irf7R	GGCATCTATGGCTTTCG GGCAAATCAGAGGGACA	qPCR of *IRF7* in *D. rerio*

## Data Availability

Sequence data reported in this study have been deposited in GenBank, accession no. OK485132.

## Supplementary Materials

The following supporting information can be downloaded at: https://www.mdpi.com/article/10.3390/v14102304/s1, Figure S1: Bioinformatic analysis of the GmIFN–γR mature peptide; Table S1: Mass Spectrometry (MS) analysis of Prokaryotic products.
