# Supplementary material for: Cloning, Exogenous Expression and Function Analysis of Interferon–γ from Gadus macrocephalus
Source: Viruses. 2022 Oct 20;14(10):2304. doi: 10.3390/v14102304 (PMC9610899; doi:10.3390/v14102304)
Supplement: Supplementary file 1 [file viruses-14-02304-s001.zip › Figure S1.pdf]

1. Sequence analysis of *GmIFN-γ receptor (GmIFN-γR)*

GmIFN-γR protein sequence was deposited to GenBank (Accession numbers OK514183).

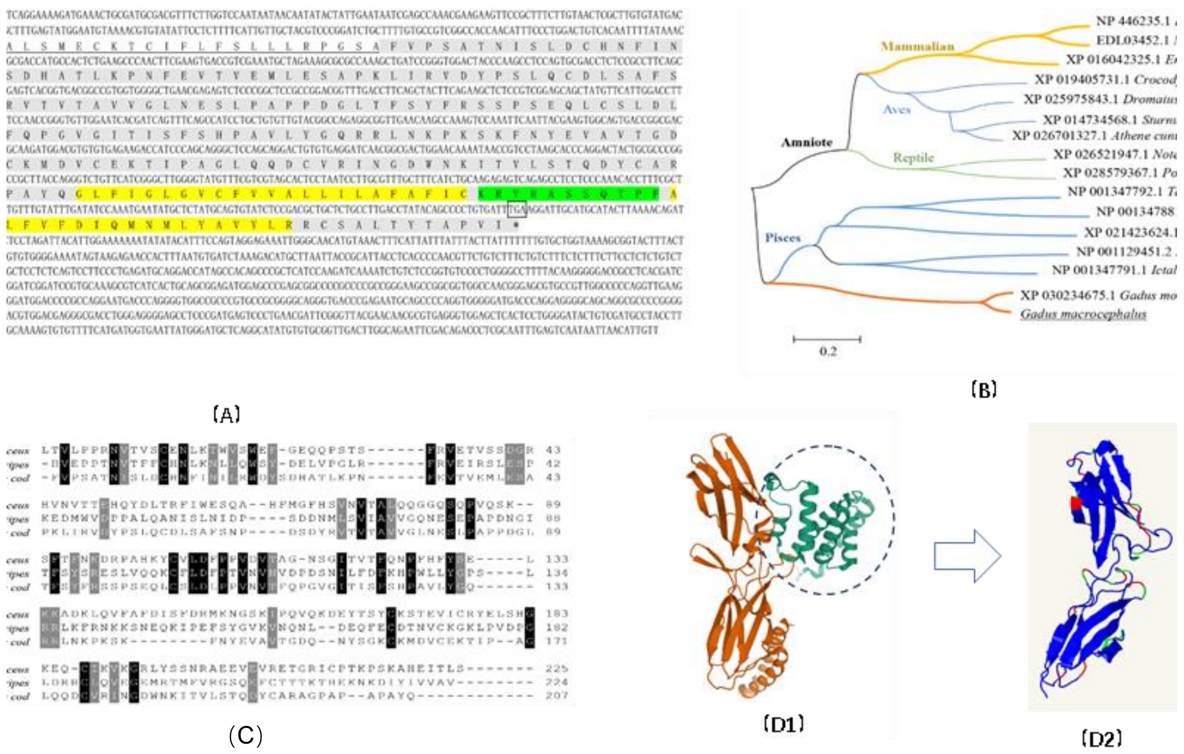

Figure S1. Bioinformatic analysis of the *GmIFN-γR* mature peptide. (A) cDNA and the deduced amino acid of *GmIFN-γR*. The signal peptide is underlined. Transmembrane regions are marked in yellow, the inside region is marked in green, and the outside is marked in gray. (B) Alignment of the outside membrane of IFN-γR from *Takifugu rubripes*, Japanese flounder, and Pacific cod. (C) Phylogeny of vertebrate IFN-γR peptides. The trees were computed using the Neighbor-Joining method in MEGA version 5.0. *Gm*IFN-γR is very similar to that of Atlantic cod, with a homology up to 87.5%, but less than 40% homology with other species. Different levels of evolution are highlighted in various color. (D1) 3-D model of mammalian IFN-γR and IFN-γ (c3besR). IFN-γR is in orange and IFN-γ is circled in green. (D2) A 3-D model of *Gm*IFN-γR was constructed based on c3besR. Secondary structure assignment was carried out using PROCHECK. Good quality is indicated in blue; poor quality in red.
