# Supplementary material for: Cloning, Exogenous Expression and Function Analysis of Interferon–γ from Gadus macrocephalus
Source: Viruses. 2022 Oct 20;14(10):2304. doi: 10.3390/v14102304 (PMC9610899; doi:10.3390/v14102304)
Supplement: Supplementary file 1 [file viruses-14-02304-s001.zip › Table S1.pdf]

Table S1. Mass Spectrometry (MS) analysis of Prokaryotic products

Location	Sequence	Mass
1-35	LPVAPVPGKMLETVKTLSLQHPQKGQSFSGSVFSR	3750.1514
36-47	ETLNKMDDGDKR	1423.6208
86-95	YLQEMVTLLR	1274.4463
158-162	AAKFR	593.6307
